# *N*-Acetylglucosamine Regulates Morphogenesis and Virulence Pathways in Fungi

**DOI:** 10.3390/jof6010008

**Published:** 2019-12-24

**Authors:** Kyunghun Min, Shamoon Naseem, James B. Konopka

**Affiliations:** Department of Microbiology and Immunology, Stony Brook University, Stony Brook, NY 11794-5222, USA; kyunghun.min@stonybrook.edu (K.M.); shamoonnaseem@hotmail.com (S.N.)

**Keywords:** *N*-acetylglucosamine, GlcNAc, *Candida albicans*, hyphal morphogenesis, *NGT1*, *HXK1*, *NAG1*, *DAC1*

## Abstract

*N*-acetylglucosamine (GlcNAc) is being increasingly recognized for its ability to stimulate cell signaling. This amino sugar is best known as a component of cell wall peptidoglycan in bacteria, cell wall chitin in fungi and parasites, exoskeletons of arthropods, and the extracellular matrix of animal cells. In addition to these structural roles, GlcNAc is now known to stimulate morphological and stress responses in a wide range of organisms. In fungi, the model organisms *Saccharomyces cerevisiae* and *Schizosaccharomyces pombe* lack the ability to respond to GlcNAc or catabolize it, so studies with the human pathogen *Candida albicans* have been providing new insights into the ability of GlcNAc to stimulate cellular responses. GlcNAc potently induces *C. albicans* to transition from budding to filamentous hyphal growth. It also promotes an epigenetic switch from White to Opaque cells, which differ in morphology, metabolism, and virulence properties. These studies have led to new discoveries, such as the identification of the first eukaryotic GlcNAc transporter. Other results have shown that GlcNAc can induce signaling in *C. albicans* in two ways. One is to act as a signaling molecule independent of its catabolism, and the other is that its catabolism can cause the alkalinization of the extracellular environment, which provides an additional stimulus to form hyphae. GlcNAc also induces the expression of virulence genes in the *C. albicans*, indicating it can influence pathogenesis. Therefore, this review will describe the recent advances in understanding the role of GlcNAc signaling pathways in regulating *C. albicans* morphogenesis and virulence.

## 1. Introduction

The ability of GlcNAc (β-d-(Acetylamino)-2-deoxy-glucopyranose) to stimulate the human fungal pathogen *Candida albicans* to form hyphae was initially reported in 1974, making it one of the first examples of this sugar acting as a signaling molecule [1]. This ability to reversibly switch growth patterns between spherical budding cells and elongated hyphal cells is a distinctive feature of *C. albicans* compared to most other fungi [2]. Morphological transitions are thought to facilitate virulence as the small budding cells are better suited to disseminate in the bloodstream and long hyphal filaments promote invasion into tissues and biofilm formation [3]. The switch to hyphal growth is also significant in that it usually coincides with the induction of virulence factors, such as adhesins, that are important for biofilm formation, superoxide dismutase to counteract oxidative stress in the host, and secreted proteases that liberate nutrients and degrade components of the host immune response [3,4,5]. A diverse set of conditions induce hyphal growth including elevated temperature (37 °C), neutral or alkaline pH of the ambient medium, CO_2_, serum, interaction with a solid extracellular matrix, and certain nutrients including GlcNAc and amino acids [2,4,6,7,8]. However, GlcNAc stands out as being one of the strongest inducers of hyphal growth.

More recently, GlcNAc was also shown to induce *C. albicans* to undergo another type of morphological switch from White phase to Opaque phase cells that were initially named for differences in colony morphology [9,10]. Most clinical isolates are White phase, but factors such as the status of the mating loci, ambient temperature, pH, CO_2_, and GlcNAc can promote a transition to the Opaque phase [3,10,11,12]. The Opaque phase cells are distinct in that they form larger, more elongated buds and express different patterns of metabolic enzymes [13]. As a result, White cells appear to be more virulent in systemic infection, but Opaque cells are better suited for commensal growth on skin [14]. Interest in White-Opaque switching has grown in recent years with the discovery that this process is regulated by an epigenetic mechanism, and that the transition to Opaque phase is needed for *C. albicans* cells to undergo mating [15,16,17].

In addition to morphological changes, GlcNAc also stimulates transcription of the genes needed for its catabolism [18,19]. The Hxk1 kinase phosphorylates GlcNAc to create GlcNAc-6-PO_4_, the Dac1 deacetylase converts it to glucosamine-6-PO_4_, and then the Nag1 deaminase converts it to fructose-6-PO_4_ which can then enter other metabolic pathways [18,20]. Subsequent studies identified the Ngt1 transporter in the plasma membrane that facilitates uptake of GlcNAc [21] As will be described in more detail below, genetic analysis of these genes has revealed that GlcNAc has to be internalized into cells in order to induce signaling, but it does not have to be metabolized as a *hxk1∆ dac1∆ nag1∆* mutant can still be induced to form hyphae [22]. However, if cells are capable of catabolizing GlcNAc, they alkalinize the extracellular medium, presumably by excreting excess nitrogen as ammonia, and this change in ambient pH synergizes with GlcNAc to help induce hyphal morphogenesis [23]. The mechanisms of GlcNAc signaling in *C. albicans* are still under investigation, but they do not appear to involve *O*-GlcNAc modification of proteins as has been observed in animal cells [24].

The significant effects of GlcNAc on *C. albicans* morphogenesis and virulence factor production have made this organism an important model for defining how fungi respond to GlcNAc. Studies on *C. albicans* have been especially important because the common model yeasts, *Saccharomyces cerevisiae* and *Schizosaccharomyces pombe*, lack the genes needed to catabolize GlcNAc and do not appear to respond to this sugar. Thus, studies in *C. albicans* have helped to better understand how GlcNAc induces cell signaling in diverse fungi, such as *Histoplasma capsulatum*, *Blastomyces dermatitidis*, *Yarrowia lipolytica*, and *Trichoderma reesei* [25,26,27]. In addition, the discovery in plants of a GlcNAc transporter homologous to Ngt1 indicated an important role for GlcNAc signaling in promoting plant–fungal interactions during arbuscular mycorrhizal symbioses [28]. Therefore, this review will summarize recent studies on GlcNAc regulation of cell signaling and morphogenesis in fungi.

## 2. GlcNAc Induces Expression of Genes Needed for Its Catabolism

**Figure 1:**
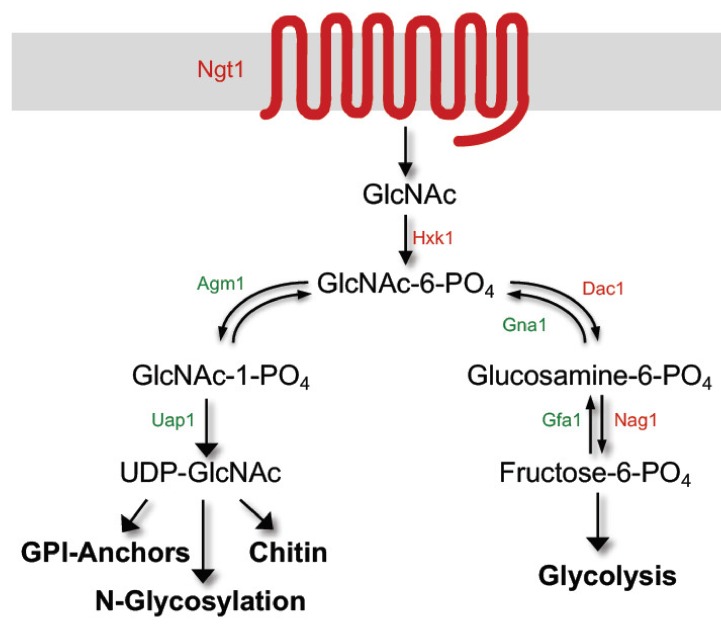
GlcNAc metabolic pathways in *Candida albicans*. Catabolic proteins are indicated in red and anabolic proteins are indicated in green. Arrows indicate the direction of the biochemical activity. GlcNAc is transported into the cell by Ngt1 and then phosphorylated by Hxk1. It can then be converted into fructose-6-PO_4_ and metabolized. Alternatively, GlcNAc-6-PO_4_ can enter the anabolic pathway and be converted into UDP-GlcNAc, a key building block of cell wall chitin, GPI-anchors on proteins, and *N*-glycosylation of proteins. Note that cells only synthesize GlcNAc-6-PO_4_ so the presence of non-phosphorylated GlcNAc can act as a unique signal for the presence of exogenous GlcNAc.

GlcNAc rapidly induces the *HXK1*, *DAC1*, and *NAG1* genes needed for its catabolism about 80-fold or more [18,19,29]. These genes are in an adjacent cluster in the genome, similar to the galactose catabolic genes [18]. Clustering highly induced genes together may help to coordinate the expected effects on chromatin structure with neighboring genes. The GlcNAc transporter gene (*NGT1*) is also highly induced [21], as is a gene of unknown function (*GIG1*) [19], which are present at different sites in the genome. In contrast, the *HEX1* gene, which encodes a secreted *N*-acetylglucosamidase that can break down GlcNAc oligomers, is only induced about 4-fold [30,31]. The pathway by which *C. albicans* catabolizes GlcNAc is generally similar to bacterial GlcNAc catabolism [32], but with some differences [33]. Ngt1 facilitates the transport of GlcNAc across the plasma membrane where it is then phosphorylated by Hxk1 to create GlcNAc-6-PO_4_, and then acted on by Dac1 and Nag1 to create fructose-6-PO_4_ (Figure 1).

GlcNAc has to be taken up by cells to induce the expression of the catabolic genes, as evidenced by the defect of *ngt1∆* mutant lacking the GlcNAc transporter in inducing the catabolic genes [21]. Some cell surface transporters can induce signaling by acting as a “transceptor” [34]. However, Ngt1 does not appear to function in this manner since it can be substituted by highly divergent GlcNAc transporters from a plant (rice) and from the distantly related fungus *Histoplasma capsulatum* [25,28]. The need for Ngt1 can also be bypassed by using high concentrations of GlcNAc that get into cells by other low affinity pathways [21].

GlcNAc metabolism does not appear to be required for induction of the catabolic genes, as an *hxk1∆* mutant can still be stimulated to induce the expression of *NAG1*, *DAC1*, and *NGT1* [22]. Similar results were observed for an *hxk1∆ dac1∆ nag1∆* triple mutant, which can still be stimulated by GlcNAc to induce expression of *NGT1*. This indicates that metabolism of GlcNAc is not required to stimulate expression of the catabolic genes, since phosphorylation of GlcNAc is required for it to enter either the catabolic or anabolic pathways (Figure 1) [32]. This also indicates that non-phosphorylated GlcNAc taken up by cells can act as a signaling molecule to stimulate cellular responses. An advantage of using non-phosphorylated GlcNAc as a signaling molecule is that cells only synthesize GlcNAc-6-PO_4_, so there should be no interference from the synthesis pathway on the induction of the catabolic pathway (Figure 1) [33,35].

GlcNAc stimulates expression of the catabolic genes by a pathway that diverges from the one that induces hyphal growth that will be described below. This is indicated by the fact that although GlcNAc can induce hyphae, other hyphal inducers such as cAMP, serum, and elevated temperature do not induce the GlcNAc catabolic genes [21,29]. Genetic screens have identified transcription factors that are important for regulation of the GlcNAc catabolic genes. It has been proposed that Ngs1, which contains a domain similar to *N*-acetylglucosaminidases, binds GlcNAc and the Ndt80-family transcription factor Rep1 recruits Ngs1 to activate GlcNAc catabolic genes [36,37,38]. There are also other layers of regulation as the expression of the GlcNAc genes is repressed in the presence of glucose [19,21].

### Open Questions

One interesting question is why GlcNAc inhibits growth of *nag1∆* and *dac1∆* mutants [22]. A similar effect was observed for the analogous mutants in bacteria, where it was thought that blocking the GlcNAc catabolic pathway resulted in too much GlcNAc being funneled into the anabolic pathway to make UDP-GlcNAc, thereby depleting UTP [39,40]. UDP-GlcNAc is used for the formation of chitin, *N*-linked glycosylation, and GPI anchors on proteins [32]. Although GlcNAc may block growth of *C. albicans nag1∆* or *dac1∆* mutants by depleting UTP and inhibiting transcription, more work needs to be done as this inhibitory effect was poorly rescued by addition of excess uridine in medium [22]. A related question involves a different mechanism of toxicity caused by incubating *C. albicans* with GlcNAc alone in water [41]. The addition of yeast nitrogen base (salts and vitamins) along with the GlcNAc rescued this toxicity, indicating it is an abnormal metabolic response. Mitochondrial mutants survived longer, suggesting this type of cell death was stimulated by aberrant respiratory metabolism [41]. A third interesting question is why the galactose catabolic genes are induced by GlcNAc, which suggests a type of metabolic crosstalk [19,23,42]. In addition, finally, it will be interesting to determine the function of *GIG1*, which is very highly induced by GlcNAc similar to the GlcNAc catabolic genes [19].

## 3. GlcNAc Stimulates a Switch to Hyphal Morphogenesis

**Figure 2:**
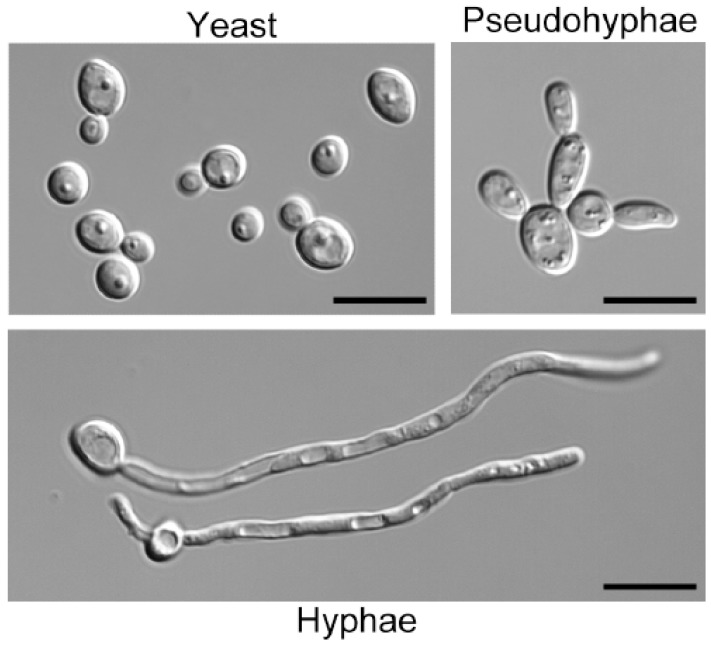
GlcNAc induction of filamentous hyphal morphology. Budding cells were grown in synthetic glucose medium to observe the yeast form, and then switched to medium containing 50 mM GlcNAc for 3 h at 37 °C to induce the transition to filamentous pseudohyphae (distinguished by pinched bud necks) and hyphal cells (chains of elongated cells with smooth parallel walls). Bars: 10 µm.

A broad range of factors stimulate *C. albicans* to switch to hyphal growth. In addition to GlcNAc these include serum, nutritional factors such as certain amino acids or low nitrogen medium, and environmental conditions including high CO_2_, alkaline pH, contact with a solid matrix, and an ambient temperature of 37 °C [2,4,6,7,8]. GlcNAc is one of the strongest inducers of hyphal growth, which has made it a useful tool for exploring the mechanisms underlying this morphological transition (Figure 2). Other amino sugars, such as glucosamine, do not induce hyphae. GlcNAc also stimulates the expression of virulence genes, such as the adhesins that promote adherence to host cells and biofilm formation [2,35].

### 3.1. GlcNAc Has to Be Taken Up by Cells to Induce Hyphal Growth

Non-phosphorylated GlcNAc appears to act as a signaling molecule to induce hyphae, as described above for the regulation of the catabolic genes, since the *hxk1∆* mutant that fails to phosphorylate GlcNAc [20] is readily induced by GlcNAc to form hyphae [22]. GlcNAc has to be taken up into the cell to induce hyphae as cells lacking the GlcNAc transporter (*ngt1∆*) require an approximately 1000-fold higher dose of GlcNAc to undergo this morphological switch [21]. Furthermore, similar to what was described above for the induction of the catabolic genes, the role of *NGT1* can be substituted by expression of a very divergent plant ortholog of *NGT1* [28], or the expression of two divergent orthologs of *NGT1* from *H. capsulatum* [25]. These results underscore an important role for increased intracellular levels of non-phosphorylated GlcNAc in transducing a signal [35]. As indicated in Figure 1, an advantage of this mechanism is that since cells are only known to synthesize a phosphorylated form of GlcNAc-6-PO_4_ [32], this provides a way for cells to distinguish elevated levels of extracellular GlcNAc from the high levels of GlcNAc-6-PO_4_ that are synthesized in cells for use as a building block in formation of cell wall chitin and other processes.

### 3.2. GlcNAc Metabolism Enhances Hyphal Gene Transcription by Alkalinizing the Extracellular Environment

The metabolism of GlcNAc is not required to induce hyphal morphogenesis, but if it is catabolized the extracellular medium becomes more alkaline, which is itself an inducer of hyphal responses [23]. The pH change is thought to occur because cells growing on GlcNAc excrete excess nitrogen as ammonia, similar to what has been reported for cells growing on amino acids [43]. This is an important factor to keep in mind when carrying out studies comparing growth on glucose versus GlcNAc, since the former results in the pH of the medium becoming more acidic whereas GlcNAc causes alkalinization. Interestingly, studies carried out with an *hxk1∆* mutant to prevent GlcNAc catabolism showed that hyphae were induced at pH 4 with little or no induction of the usual hyphal-induced genes [23]. However, induction of hyphal genes was restored if the pH was buffered closer to neutral pH. Further studies showed that a *rim101*∆ mutant that is defective in sensing alkaline pH was partially defective in inducing hyphal genes. This indicates that GlcNAc requires a second signal from the ambient pH to fully induce hyphal genes. It has also been suggested that catabolism of GlcNAc could cause amino acid depletion that stimulates hyphal growth, which might also contribute in a synergistic manner to hyphal signaling [44,45].

### 3.3. GlcNAc Induces a cAMP-Independent Signal

**Figure 3:**
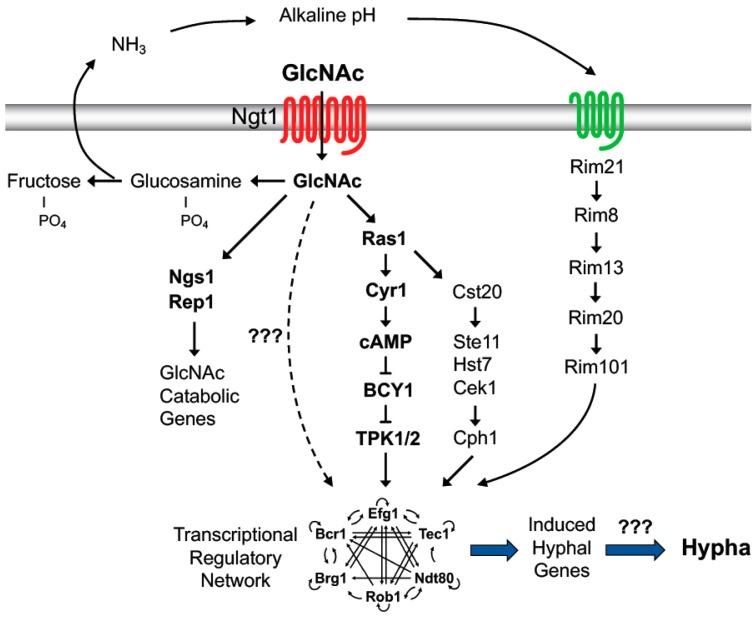
Signal transduction pathways activated by GlcNAc. GlcNAc induces a pathway that acts through the Ngs1 and Rep1 transcription factors to induce the GlcNAc catabolic genes. It also induces a cAMP-independent pathway to stimulate hyphal growth (dashed line). These pathways do not require metabolism of GlcNAc. However, if GlcNAc is catabolized it makes the extracellular environment more alkaline, which activates signaling through the Rim101 pathway that synergizes with GlcNAc to stimulate hyphal morphogenesis and gene induction. The wavy green line in the plasma membrane indicates the pH sensing complex. Lines with an arrowhead indicate activation and lines with a blunt tip indicate negative regulation.

Most current models propose that hyphal inducers stimulate adenylyl cyclase to form a spike in cAMP levels that promotes the transition to filamentous hyphal growth [2,3,46]. In support of this, adding high levels of cAMP to cells can induce hyphal morphogenesis [44,47,48,49,50] and a *cyr1∆* mutant that lacks adenylyl cyclase is defective in forming hyphae in response to a broad range of conditions, including GlcNAc [44]. However, some stimuli, such as GlcNAc, do not cause a spike in cAMP [51]. A more serious complicating factor is that the *cyr1∆* mutant grows very poorly and has altered expression of many different kinds of metabolic genes [44]. It was therefore interesting that faster-growing pseudorevertants of a *cyr1∆* strain could be induced to form hyphae even though they lack adenylyl cyclase and cAMP [52]. This indicates that GlcNAc can stimulate a signal pathway to induce hyphae that is independent of cAMP (Figure 3).

### 3.4. Hyphal-Induced Genes Do Not Play an Obvious Role in Hyphal Morphogenesis

It has been assumed that GlcNAc and other hyphal inducers act by stimulating gene expression because deletion of a repressor (e.g., *NRG1*) or overexpression of a transcriptional activator (e.g., *UME6*) can induce hyphae [2,3,53,54,55,56]. The mechanisms that regulate hyphal-induced gene expression have been reviewed in more detail elsewhere [3,57]. However, in spite of many different transcriptomic studies, the key target genes have not been identified. One challenge is that the extent of hyphal gene induction does not correlate with the switch to hyphal growth. For example, the common core set of genes stimulated by a range of different hyphal inducing conditions only identified eight genes, none of which appear to be responsible for stimulating hyphal growth [58]. In addition, as described above, a mutant that cannot catabolize GlcNAc can be stimulated by GlcNAc to form hyphae without obvious induction of hyphal-specific genes [23]. Other studies have shown that some transcription key factors needed for hyphal growth can be bypassed under special conditions [59,60,61]. Thus, although it is clear that transcriptional regulation is required to make cells competent to be induced to form hyphae, there is no clear evidence at this point that the hyphal-induced genes promote hyphal growth, which has led to development of alternative models, such as translational regulation [57].

### 3.5. Protein Phosphorylation Promotes Hyphal Morphogenesis

Protein phosphorylation has been strongly implicated in the regulation of hyphal growth, but more work needs to be done to better define the mechanisms. Several protein kinases contribute to hyphal growth including the cAMP-dependent PKA, the Cek1 MAP kinase, and the Hgc1-Cdc28 cyclin-dependent kinase [2,3]. The Hgc1-Cdc28 kinase is strongly implicated as it appears to phosphorylate multiple proteins that influence hyphal growth [62]. These include Exo84, Rga1, Sla1, Sec2, Mob2, Efg1, and the septins Sep7 and Cdc11 [62,63,64,65,66,67,68,69,70]. The *cyr1∆* and *efg1∆* mutants express very low basal levels of *HGC1* [54], which likely contributes to their failure to form hyphae. However, *HGC1* overexpression is not sufficient to induce hyphae [71], and an *hgc1∆* mutant can still initiate hyphae, although it cannot maintain highly polarized hyphal morphogenesis and forms broad outgrowths instead [71]. Thus, further work needs to be carried out to fully identify the role of phosphorylation in regulation of hyphal morphogenesis [72].

### 3.6. Open Questions

One important question is how the different hyphal inducers stimulate overlapping, but distinct patterns of gene expression, and what role the target genes play, if any, in hyphal morphogenesis [57,58]. Another interesting question is what is the cAMP-independent pathway for GlcNAc induction of hyphae [52]? It will also be important to determine the source of GlcNAc in vivo that can stimulate *C. albicans* to form hyphae. There are a several possibilities, but one major source is likely to be the extracellular surface as GlcNAc is an important component of bacterial peptidoglycan, fungal cell wall chitin, and is very abundant in the extracellular matrix glycosaminoglycans of animal cells [73,74].

## 4. GlcNAc Induces the White-Opaque Epigenetic Switch

*C. albicans* can also undergo another type of morphological transition known as White-Opaque switching that was first described in a clinical isolate of *C. albicans* [9]. The White and Opaque phase cells were named after the appearance of their colonies on agar medium [9]. Opaque cells are morphologically distinct from White cells as they are more elongated and are approximately three-times larger by volume. The surface of the cell wall of Opaque cells when viewed by scanning electron microscopy exhibits a unique pimpled pattern with an unknown biological role [75]. The different cell types are functionally distinct and have very different metabolic profiles [13]. White cells are more virulent in mouse bloodstream infection models [14,76,77]. Opaque cells have been reported to colonize skin more effectively in a neonatal mouse skin infection model [14,76] and are more resistant to phagocytosis [78] and grow faster in an ex vivo tongue infection model [77]. Interest in White-Opaque switching was greatly enhanced by the discovery that mating between opposite mating type strains (MTL strains a/a and α/α) increased dramatically after a switch to Opaque phase [16]. White-Opaque switching is regulated in part by epigenetic mechanisms [3]. Although studies on the regulation of switching have gained attention as model for epigenetic regulation, this complex form of transcriptional regulation is beyond the scope of this review.

### 4.1. GlcNAc Regulates White-Opaque Switching

Similar to the transition to hyphal morphogenesis, White-Opaque switching is highly sensitive to environmental conditions. It can be induced by conditions such as GlcNAc, >5% CO_2_, and acidic pH, whereas glucose, low levels of CO_2_, alkaline pH, and human body temperature (37 °C) promote the switch back to the White state [9,10,11,12]. GlcNAc is an interesting regulator of this transition because of its ability to stimulate the White to Opaque switch in cells that are heterozygous at the mating loci (a/α) [76]. The regulators a1 and α2 encoded at the respective *MTL* mating loci form a heterodimeric transcription factor that usually prevents White-Opaque switching in a/α cells by direct transcriptional repression of genes (such as *WOR1*) that are required for the switch [79,80,81]. However, Xie et al. found that approximately one third of a/α clinical isolates from a collection of patient samples were capable of switching from White to Opaque when cultured on GlcNAc medium at 25 °C in 5% CO_2_ [76]. Opaque cells of a/α strains exhibit similar features of cellular and colony morphology to their MTL homozygous counterparts but were incapable of mating. Thus, although switching is normally blocked in a/α cells, this barrier can be overcome by synergy of GlcNAc and CO_2_.

GlcNAc is thought to promote White/Opaque switching primarily through activation of the cAMP signal pathway and subsequent phosphorylation of a key transcriptional regulator, Wor1 [10]. The transcriptional factors Rfg1, Brg1, Sfl2, and Efg1 control the expression of WOR1, which then determines the White or Opaque state [76,82]. Recent studies discovered that GlcNAc does not have to be metabolized to induce the switch to Opaque [83,84]. Interestingly, deletion of *HXK1* induced the Opaque phenotypes in the SC5314 strain (a/α) [83,84]. However, deletion of other GlcNAc catabolic genes *NGT1*, *NAG1*, and *DAC1* in the same background strain had no obvious effect on switching [83]. Although these results could suggest that Hxk1 may play special roles in regulation of Opaque switching, the induction of the Opaque phenotype in the *hxk1∆* mutant is likely a response to the intracellular accumulation of GlcNAc liberated during the remodeling of cell wall chitin or degradation of glycosylated proteins. A similar phenomenon was previously reported for the ability of an *hxk1∆* mutant to spontaneously form hyphae [22]. The autoinduction of hyphae in the *hxk1∆* mutant was attributed to the buildup of GlcNAc since it was only seen when cells were grown to high cell density and not when they were kept at low cell density.

### 4.2. Open Questions

There are still many questions to sort out in terms of how GlcNAc influences the molecular mechanisms that regulate White-Opaque switching. It will also be important to determine how GlcNAc influences other types of phenotypic transitions seen in *C. albicans* such as the Gray, GUT (Gastrointestinally-IndUced Transition), and INT (Intermediate) phenotypes that share overlapping mechanisms of regulation with White-Opaque switching [77,85,86].

## 5. Roles of GlcNAc in *C. albicans* Commensalism and Virulence

### 5.1. GlcNAc Influences Virulence Functions

*C. albicans* cells exposed to GlcNAc induce many genes that are important for virulence [19,23,29]. These genes encode functions such as adhesin proteins that promote attachment to host cells and biofilm formation, secreted superoxide dismutases that counteract the oxidative burst in the host, secreted aspartyl proteases that liberate nutrients and degrade host immune system factors, and Candidalysin, which forms pores in host cell plasma membrane [3,23,87,88]. These results have suggested that GlcNAc plays a role in virulence.

One site where GlcNAc has been implicated as having a role in vivo is in surviving phagocytosis, as the ability to metabolize GlcNAc promotes the growth of *C. albicans* in the macrophage phagosome [74]. GlcNAc can function in two ways in the phagosome. It can act as a signaling molecule to help induce virulence factors, such as the secreted superoxide dismutases Sod4 and Sod5 that can counteract the oxidative burst in phagosome [89]. It can also induce hyphal growth, which can damage the phagosomal membrane and promote alkalinization of the lumen [90]. The other way GlcNAc can act is that catabolism of GlcNAc and the subsequent release of excess nitrogen as ammonia can counteract the acidification of the phagosome [74]. Interestingly, growth on GlcNAc and other non-glucose carbon sources, such as amino acids and lactate, promotes resistance to various kinds of stress that contribute in an additive manner to surviving attack from host defense mechanisms [91].

GlcNAc has also been implicated in promoting systemic candidiasis, since the GlcNAc catabolic mutants *nag1∆*, *dac1∆*, and *hxk1∆* are defective in virulence [20,92]. However, these studies are complicated by the fact that the growth of the *nag1∆* and *dac1∆* mutants is inhibited by GlcNAc [22]. A triple *hxk1∆ nag1∆ dac1∆* mutant is not inhibited by GlcNAc and is defective in virulence [23]. However, this strain is also complicated in that it spontaneously forms hyphae and has altered cell wall structures, which are thought to be caused by these cells responding in an autocrine manner to GlcNAc released during cell wall remodeling that cannot be metabolized in the mutant cells [22,93]. In contrast, an *ngt1∆* mutant that lacks the GlcNAc transporter was not defective in virulence [23]. Altogether, these results indicate that the GlcNAc catabolic genes are required for normal function of *C. albicans* in the host, but that the ability to respond to exogenous GlcNAc or metabolize it is not required for systemic candidiasis.

### 5.2. Potential Roles of GlcNAc in Commensalism

GlcNAc has been suggested to play a role in colonization of the gastrointestinal (GI) tract where *C. albicans* typically resides as a commensal organism. GUT (Gastrointestinally-IndUced Transition) phenotype cells that were isolated from the GI tract of mice had elevated expression of some GlcNAc genes [85]. However, there does not appear to be an important role for GlcNAc catabolism to induce hyphae in the GI tract. One reason is that *efg1*∆ mutants that are defective in forming hyphae are hypercompetitive in the GI tract [86,94]. Further studies indicated that it is the induction of the virulence genes, particularly the SAP secreted aspartyl proteases, rather than hyphal morphology, that decreases the ability of wild type *EFG1* strains to compete for growth in the GI tract [95].

### 5.3. Open Questions

The role of GlcNAc in *C. albicans* virulence is still an open question as the mouse studies described above primarily test for role of GlcNAc in the kidney, the main target organ of *C. albicans* in the mouse. In humans, *C. albicans* infects a very broad range of tissues where GlcNAc might be more critical. In this regard it is interesting that the ability to catabolize GlcNAc is needed for virulence of the human parasite *Leishmania* [96]. It is not clear how many sites there are in the body where GlcNAc would be readily available in addition to the phagosome, but it is a major component of glycosaminoglycans in the extracellular matrix, and *N*-linked glycosylation of proteins. In addition to affecting cell signaling, it is also possible that growth of cells on GlcNAc will lead to altered levels of *N*-glycosylation and chitin at the cell surface that could influence interaction with the host immune system [97,98]. Thus, GlcNAc may not be essential for all types of candidiasis, but it is likely an important part of broader metabolic flexibility that makes *C. albicans* able to infect such a broad range of tissues.

## 6. Roles of GlcNAc in Other Fungal Species

### 6.1. GlcNAc Catabolism in Other Fungal Species

Although most fungal species carry the genes required to catabolize GlcNAc, it is not a requirement for success in the environment or for pathogenesis, since the genome sequences of many species lack orthologs of the GlcNAc catabolic genes. For example, *S. cerevisiae* lacks the GlcNAc catabolic genes as does the somewhat closely related plant pathogen *Ashbya gossypii* and the human pathogen *Candida glabrata*. Furthermore, the fission yeast *S. pombe* and its closest relative that is a human pathogen, *Pneumocystis jiroveci*, also lack the GlcNAc catabolic genes. Furthermore, some species are unable to utilize GlcNAc because they carry mutations in the GlcNAc genes. For example, *Neurospora crassa* carries a mutation in the ortholog of *NAG1* that encodes the glucosamine-6-PO_4_ deaminase [99]. In addition, the human pathogen *Candida africana* carries a mutation in the *HXK1* gene and is therefore unable to phosphorylate GlcNAc to allow it to enter the metabolic pathways, although it can still respond to exogenous GlcNAc [100]. Thus, although the ability to utilize the common sugar GlcNAc as a source of carbon and nitrogen is thought to be beneficial to many species, it is not essential for virulence.

### 6.2. GlcNAc Can Stimulate or Inhibit Hyphal Morphogenesis in Other Species

GlcNAc has been reported to stimulate hyphal morphogenesis in other species that transition between yeast and filamentous growth forms, such as *Yarrowia lipolytica* [26,101]. For the thermally dimorphic human fungal pathogens, *Histoplasma capsulatum* and *Blastomyces dermatitidis*, which grow as yeast at 37 °C in the host and as filamentous hyphae at lower temperatures in the environment, GlcNAc promotes a more rapid return to hyphal growth after a shift to lower temperature [25]. In contrast, GlcNAc has been shown to inhibit hyphal growth in *Candida tropicalis*, although GlcNAc was still able to induce the White to Opaque switch [102,103], and it was not able to induce true hyphae in *Candida dubliniensis* [104]. It is also interesting that glucosamine, but not GlcNAc, can stimulate filamentous growth of *Cryptococcus neoformans* [105].

### 6.3. GlcNAc Roles in Interspecies Communication

The ability of GlcNAc to act as a signaling molecule suggests that it may also transduce interspecies communication. This possibility is strengthened by discovery that a plant ortholog of the Ngt1 GlcNAc transporter mediates arbuscular mycorrhizal symbiosis [28]. Land plants typically form a mutualistic symbiosis with arbuscular mycorrhizal fungi. The plants provide sugar to the fungi and the fungi provide nutrients from the soil, thereby enabling both organisms to grow better. This is a complex process in which the two species signal each other to guide the stages of root colonization by the fungus, which results in the fungus penetrating into plant cells [106,107]. Interestingly, a genetic screen in maize identified a mutant that could not interact with mycorrhizal fungi, which was termed *nope1* for “no perception” [28]. Genetic mapping revealed that the plant *NOPE1* encoded an ortholog of *NGT1*, and its ability to transport GlcNAc was confirmed in part by showing that *NOPE1* could rescue a *C. albicans ngt1Δ* mutant for ability to take up GlcNAc and form hyphae in response to this sugar [28]. Other studies showed that GlcNAc genes are induced in the fungal partner during arbuscular mycorrhizal symbiosis [108]. Interestingly, small GlcNAc-containing molecules also play roles in signaling via LysM domain proteins to induce symbiosis and or warn of a potential pathogen [109].

### 6.4. Open Questions

An interesting question is whether GlcNAc signaling in other species is due to GlcNAc acting as a signaling molecule, or whether it is alkalinization of the extracellular environment caused by GlcNAc catabolism that promotes a signal. It is also interesting that many species have lost the ability to catabolize GlcNAc. It is not clear if there are advantages under some conditions to avoid utilizing GlcNAc, or if there is just no strong selective pressure against it in some organisms. In this regard it is interesting that the GlcNAc catabolic genes are present in an adjacent cluster in most fungal species, making it less complicated for cells to lose all three genes [27,110].

## 7. Future Directions

The mechanisms by which GlcNAc can act as both a nutrient and a signaling molecule in *C. albicans* provide a model for ways in which this sugar can promote cellular responses in other organisms. GlcNAc may be similar to the various chemical messengers and quorum factors that are used for both intra and interspecies communication [111]. One interesting possibility is that since GlcNAc is part of the cell wall or extracellular matrix of bacteria, fungi, parasites, and animals, the release of GlcNAc may be a universal signal of cell damage due to attack by a pathogen, or it could also be an indicator of actively growing bacteria nearby, since cell wall remodeling releases significant amounts of GlcNAc [112]. This possibility is supported by the ability of GlcNAc to regulate virulence functions and stress resistance factors in fungi and bacteria [113]. Furthermore, GlcNAc can stimulate NLRP3 inflammasome activation in mammalian cells, which leads to processing and secretion of interleukin (IL)-1β and IL-18 [114]. GlcNAc is also well suited to promote symbiotic interactions between microbes and a mammalian host, analogous to what has been seen with plants and fungi [28,109]. It will therefore be interesting for future studies to define how GlcNAc signaling contributes to cell–cell communication in biologically diverse niches, such as the human gut or polymicrobial infections, that contain a mix of bacteria, fungi, and human cells.
